# Diastasis plication during robotic transabdominal preperitoneal repair (rTAPP): a case series of outcomes at a tertiary care center

**DOI:** 10.1007/s00464-025-11989-7

**Published:** 2025-07-18

**Authors:** Alex Jog, Leah J. Schoel, Desmond Huynh, Matt L. Kirkland, Anne P. Ehlers, Dana A. Telem, Jenny M. Shao

**Affiliations:** 1 University of Michigan Medical School, Ann Arbor, MI USA; 2 Department of Surgery, University of Michigan, Ann Arbor, MI 48109 USA; 3 Department of Surgery, University of Pennsylvania, Philadelphia, PA USA

**Keywords:** Ventral hernia, Rectus diastasis, Robotic hernia repair, Diastasis plication, Hernia recurrence

## Abstract

**Background:**

Despite increasing popularity, minimal data exist regarding diastasis plication during robotic ventral hernia repair for small defect sizes. We evaluated the safety and short-term efficacy of this procedure in a series of 30 patients at a tertiary care center using a single robotic technique.

**Methods:**

We performed a single-institution retrospective study of adult patients who underwent elective robotic ventral hernia repair and concomitant diastasis plication with mesh between 10/1/2020 and 12/31/2022. Patients were identified from the electronic medical record and clinical and operative data were manually abstracted from patient charts.

**Results:**

Thirty patients underwent robotic transabdominal preperitoneal repair and diastasis plication with mesh for hernia defects < 5 cm. Patient characteristics included mean age 50.6 ± 11.4 years, 50% female, BMI 32.5 ± 6.5 kg/m^2^, 6.7% DMII, and 23.3% active smokers. Median operative time was 113.5 (IQR 97–147.5) minutes with 22% of patients undergoing a concurrent hernia repair (spigelian, inguinal, or multiple separate midline defects). Median hernia defect width was 3 ± 1.4 cm, median diastasis length was 10 ± 3.7 cm, and median mesh area was 64 ± 52.7 cm^2^. No patients experienced surgical site infection, mesh infection, seroma, hematoma, or reoperation. Within 30 days, 3 patients were seen in the emergency department for superficial thrombophlebitis, reflux, and pain/emesis. There were no patients with clinical or radiographic recurrence at a mean postoperative follow-up of 116.0 ± 117.0 days.

**Conclusions:**

Routine diastasis plication during robotic ventral hernia repair is safe and effective, with no increased rates of complications or adverse events and with no documented clinical or radiographic recurrence.

Anterior abdominal wall (ventral) hernias often present with concomitant rectus diastasis [1]. Uncorrected rectus diastasis has been established as a risk factor for hernia recurrence given the poor quality of tissue surrounding the diastasis defect, which can impede the long-term effectiveness and durability of the hernia repair [2]. Furthermore, rectus diastasis is associated with increasing age, obesity, and multiparity [1]. Given the high risk of potential recurrence with an unaddressed diastasis at the time of initial hernia repair, understanding rectus diastasis and optimizing surgical management are increasingly salient concerns to avoid postoperative complications and recurrence in this high-risk population.

Despite the clinical significance of rectus diastasis, limited data exist regarding optimal surgical approach, and there is no defined standard of care in hernia guidelines. Current guidelines demonstrate a general lack of consensus for diastasis management. While the International Endohernia Society (IEHS) supports routine diastasis plication during ventral hernia repair, the European (EHS) and American (AHS) Hernia Societies conclude that simultaneous rectus diastasis repair is “optional” with insufficient evidence to recommend any particular approach to repairing concomitant rectus diastasis and ventral hernia [3, 4]. Advances in robotic surgery now enable general surgeons to perform diastasis plication during hernia repair through the same small incisions with minimal increase in operative time and without a separate procedure performed by a plastic surgeon [5]. The rapid uptake of robotics utilization among hernia surgeons thus provides a timely opportunity to evaluate the efficacy of robotic techniques for concurrent ventral hernia and diastasis repair.

While prior studies have demonstrated a low rate of short-term complications and readmissions for concurrent ventral hernia and diastasis repair, long-term outcomes including hernia recurrence for patients undergoing robotic repair of a ventral hernia with rectus diastasis remain unknown [6]. Within this context, we examined outcomes for 30 patients undergoing robotic transabdominal preperitoneal repair (rTAPP) for the surgical management of small ventral hernias (< 5 cm) with concurrent rectus diastasis plication at a single site. Our primary outcome was efficacy, assessed by clinical and radiographic hernia recurrence. Secondary outcomes included adverse postoperative outcomes, complications, and readmissions.

## Methods

### Study design and data source

Following approval by the University of Pennsylvania Institutional Review Board, a retrospective review of cases was conducted to evaluate rTAPP with diastasis plication performed at a tertiary care center between 10/1/2020 and 12/31/2022. Inclusion criteria included patients > 18 years of age undergoing rTAPP procedure with a hernia size of < 5 cm in diameter, concurrent rectus diastasis plication, and intraoperative mesh placement. Exclusion criteria included emergent operations and larger hernia defects that needed more extensive dissection or a retrorectus approach. Patient demographics, hernia and operative details, and all available follow-up data were manually abstracted from patient charts.

### Operative technique

**Fig. 1:**
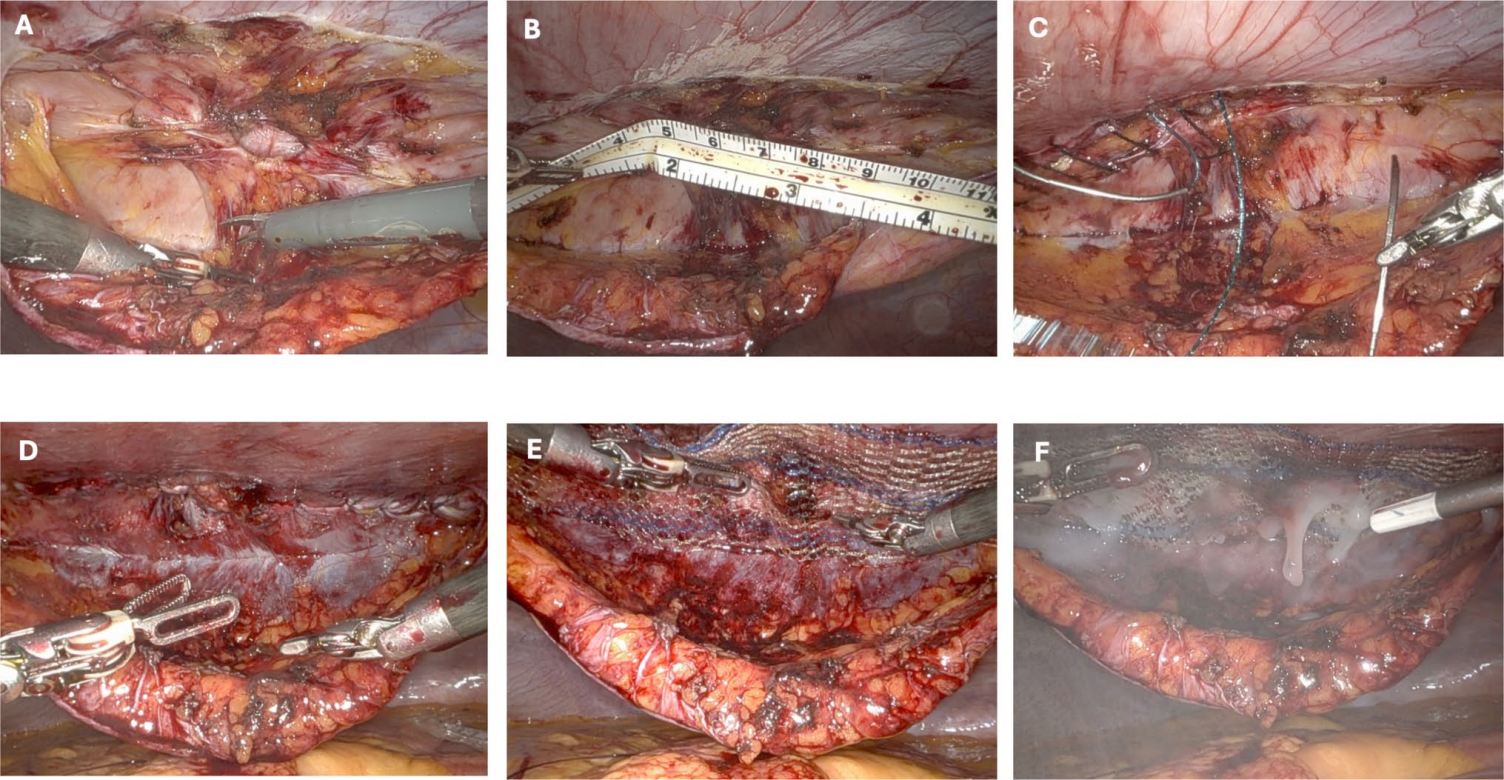
Robotic transabdominal preperitoneal ventral hernia repair with diastasis plication. **A**. Preperitoneal flap dissection **B**. Measurement of fascial defect and diastasis **C**. Plication of diastasis and fascial defect closure **D**. Completed fascial defect closure and rectus reapproximation **E**. Mesh placement **F**. Fibrin glue fixation of mesh

All patients underwent a similar operative technique with a robotic transabdominal preperitoneal dissection (Fig. 1). After insufflation to the intraabdominal cavity usually with veress access at Palmer’s point, direct optiview access using an 8-mm robotic trocar was utilized to access the abdomen in the left upper quadrant at the site of the upper lateral port. Then 2 additional 8-mm robotic ports are placed. Using the robotic monopolar scissors and fenestrated bipolar, the preperitoneal plane was accessed by starting at the preperitoneal fat plane and taking this off of the anterior abdominal wall. This flap dissection extends superiorly and interiorly from the hernia site, and the hernia sac is reduced as a part of this repair. Concurrent diastasis of the abdominal wall is exposed in this preperitoneal plane—majority of which extends superiorly from the umbilicus. This dissection is taken up superiorly toward the xiphoid. Once the dissection plane is completed, a 0 slowly absorbing barbed suture is used to close the fascial defect and plicate the diastasis to approximate the anterior abdominal wall. If the hernia defect is at the umbilicus, the umbilical stalk is imbricated and tacked down and incorporated in the fascial defect closure. The plicating suture is done for the diastasis to reapproximate the diversification using an inverting stitch to decrease midline ridge formation. Once the rectus muscles are approximated, the preperitoneal flap space is measured and a midweight large-pore non-coated polypropylene mesh is placed directly against the anterior abdominal wall and fixated with fibrin glue. The flap is then closed with absorbable 2–0 barbed suture using a Connell stitch to exclude the mesh from the abdominal cavity. A transversus abdominis plane block is completed under direct visualization for postoperative pain control.

### Demographic and outcome variables

Demographic data included patient age, sex, BMI, comorbidities (type II diabetes, hypertension, hyperlipidemia, COPD, and history of malignancy), smoking status, and ASA score. Operative data included hernia defect dimensions, mesh dimensions, duration of procedure, and presence and type of concurrent separate site hernia repair. The primary outcome assessed was recurrence, defined as clinical or radiographic recurrence. Radiographic recurrence was documented based on a radiologist’s assessment of the most recent available imaging study. Clinical recurrence was assessed by physical exam performed by the operating surgeon on follow-up. Secondary outcomes included 30-day readmissions and ED visits, and complications including surgical site infections. All cases with a concurrent separate site hernia repair were compared to cases without a concurrent operation.

### Data analysis

Data were analyzed using STATA Version 17 (StataCorp, College Station TX, USA). Descriptive statistics were used to characterize the central tendency and distribution of quantitative variables across patient characteristics, intraoperative characteristics, and postoperative outcomes. Two-sample t-tests were performed to compare outcomes of patients with and without concurrent operation at a separate site with significance defined as *p* < 0.05.

## Results

During this period, a total of 30 patients were identified who underwent robotic transabdominal preperitoneal repair (rTAPP) and diastasis plication with mesh for hernia defects < 5 cm (Table 1). The patient cohort included 15 female patients and 15 male patients with a mean age at time of operation of 50.6 ± 11.4 years. Baseline patient characteristics included a mean BMI of 32.1 ± 6.0 kg/m^2^ and median ASA score of 2 with an interquartile range of 2–3. Prior to surgery, 14 patients had hypertension (46.7%), 15 had hyperlipidemia (50%), 2 had prior malignancy (6.7%), and no patients had COPD. Active smoking was reported by 7 patients (23.3%). Four patients (13.3%) had received prior hernia surgery: three had a prior repair of a ventral hernia, and one patient had a prior repair of an inguinal hernia. The three patients with previous ventral hernia repair were undergoing reoperation for a recurrence of the same defect.

**Table 1:** Baseline demographics (*n* = 30)

Mean age (SD)	50.6 ± 11.4 years
Mean BMI (SD)	32.1 ± 6.0 kg/m^2^
Median ASA (IQR)	2 (2–3)
Sex, male	15 (50%)
Comorbidities	
Type 2 diabetes	2 (6.7%)
Hypertension	14 (46.7%)
Hyperlipidemia	15 (50%)
COPD	0 (0%)
Prior malignancy	2 (6.7%)
Active smoking	7 (23.3%)
Previous hernia repair	4 (13.3%)
Same site	3 (75%)
Alternate site	1 (25%)

Operative details (Table 2) demonstrated a median hernia width of 3 ± 1.4 cm along the longest side of the defect and median hernia area was 8.25 cm^2^. Median diastasis length was 10 ± 3.7 cm. Mesh was used in all repairs with a mean median mesh area of 64 ± 52.7 cm^2^. Mesh was placed in a preperitoneal location in all cases. The median operative time was 113.5 min (IQR 97–147.5). Seven patients (23.3%) in this cohort underwent concurrent surgery at a separate site. These cases included six separate site hernia repairs performed by the same surgeon and one bilateral salpingo-oophorectomy performed by a gynecologic surgeon. All concurrent operations were planned in advance. Cases involving concurrent separate site operations were compared to cases without concurrent operation. No significant difference was observed across mean hernia defect width, diastasis length, or mesh area but significantly longer operative times were observed for concurrent repairs, with an average increase of 37.8 min.

**Table 2:** Operative characteristics (*n* = 30)

Median hernia defect width (SD)	3 ± 1.4 cm
Median diastasis length (SD)	10 ± 3.7 cm
Median mesh area (SD)	64 ± 52.7 cm^2^
Median operative time (IQR)	113.5 (97–147.5) minutes
Concurrent separate site operation	7 (23.3%)
Separate site hernia repair	6 (85.7%)
Other procedure*	1 (14.3%)

^*^Bilateral salpingo-oophorectomy

Postoperative outcomes demonstrated minimal complications and no recurrences (Table 3). Patients were followed by the operating surgeon for a mean duration of 116 ± 117 days, with a maximum of 433 days. Across this follow-up period, patients were monitored for short-term (30-day) and long-term (up to last date of follow-up) complications. Within 30 days, there were no readmissions. Four emergency department visits within 30 days were documented: three were for concerns of dysphagia, continued pain, and transient ischemic attack, and one for a psychiatric condition diagnosed prior to the time of surgery. There were no reported instances of surgical site infection, mesh infection, seroma, or hematoma. During the entire follow-up period, no patients required reoperation or reintervention. There were no instances of radiographic or clinical recurrence for any patients in the cohort across all available follow-up data.

**Table 3:** Postoperative outcomes (*n* = 30)

30-day outcomes	
Readmission	0
ED visits	3 (10%)
Surgical site infection	0
Mesh infection	7 (23.3%)
Seroma	6 (85.7%)
Hematoma	1 (14.3%)
Mean follow-up duration (SD)	116 ± 117 days
Clinical recurrence	0
Radiographic recurrence	0

## Discussion

In this single-center study, we examined a series of 30 patients who underwent elective robotic transabdominal preperitoneal repair (rTAPP) for small (< 5 cm in diameter) ventral hernias with concurrent rectus diastasis plication. This study had two principal findings. First, we demonstrate that this procedure is safe, with minimal increase in operative time and equivalent postoperative complications and short-term adverse outcomes to rTAPP with just closure of the fascial defect. Second, our data provide preliminary evidence for the effectiveness of this procedure, with no clinical or radiographic recurrences observed at long-term follow-up. These findings support routine diastasis plication during robotic ventral hernia repair as there are minimal downsides.

The rapid uptake of robotic surgery over the past decade provides an important context in which to reevaluate optimal surgical management of ventral hernias with concomitant rectus diastasis [7, 8]. Previously during primarily open repairs, plications were less likely to be done due to the length of the incision needed or extensive flap dissection required. The ability of a minimally invasive repair to enable surgeons to suture and plicate diastasis at the same time as hernia repair using the same incisions with minimal additional operative time and complication risk poses a new question—should this now become routine practice in patients who have concurrent diastasis and ventral hernias? Other minimally invasive techniques for concurrent diastasis plication and ventral hernia repair including the SubCutaneous OnLay endoscopic Approach (SCOLA) have also been described, but because this technique utilizes onlay mesh with a large subcutaneous dissection plane, there may be higher wound risks in patients with higher BMI and additional comorbidities [9]. Therefore, a rTAPP approach to concurrent diastasis and ventral hernia repair may be more widely applicable to the most patients, especially those who are higher risk, by employing the advantages of both techniques by using a minimally invasive technique that has low wound morbidity, while decreasing hernia recurrence.

Postoperative outcomes following rTAPP with diastasis plication were found to be in-line with those reported by previous analyses of robotic ventral hernia repair both with and without concomitant diastasis. Tran et al., in a meta-analysis of 10,273 robotic ventral hernia cases across 39 studies, reported a mean rate of surgical site infection (SSI) of 1.22% within 30 days, surgical site occurrences (SSO) of 14.11% within 30 days, a 30-day readmission rate of 3.98%, and 30-day reoperation rate of 0.68% [10]. In comparison, a retrospective study by de Figueiredo et al. found a 2% readmission rate and 4% incidence of SSO within 30 days for patients undergoing combined repair of ventral hernia and rectus diastasis in a cohort that included both open and minimally invasive operations [6]. Our analysis reports similarly low rates of SSI, readmissions, and reoperations within 30 days with no cases of seroma or hematoma postoperatively in a cohort of entirely robotic cases. Further support for the relative safety of combined diastasis repair and robotic ventral hernia repair is offered by Jaro et al., who find no significant difference in short-term morbidity between patients who underwent diastasis repair and those who did not during robotic ventral hernia repair performed at a single institution [5]. The cosmetic outcomes after a robotic diastasis plication and hernia repair are not well reported in the literature. Based on the author’s experience, for small hernia defect sizes and diastasis plication of less than 3 cm, generally patients do not develop a noticeable visual ridge after surgery, especially if their BMI is > 30 kg/m^2^. For a small subset of patients if a visual ridge is seen, it typically resolves several weeks after surgery. Collectively, our findings indicate that the safety profile of combined robotic ventral hernia repair and diastasis plication is similar to that of robotic ventral hernia repair alone, suggesting that the addition of the plication procedure may not contribute to excess morbidity within 30 days.

In addition to evaluating the safety of combined rTAPP and diastasis plication, our study provides an assessment of the efficacy of this procedure. We observed no cases of clinical or radiographic recurrence within a mean follow-up duration of 116 days. This low rate of recurrence is similar to rates reported by prior studies that have evaluated all patients undergoing robotic ventral hernia repair. Fry et al., in an analysis of 12,693 robotic cases in Medicare claims data, provided a model that estimated recurrence risk at less than 1% within 6 months (182 days) [11]. However, as Fry et al. demonstrate, recurrence risk increases with time, with a 13.43% rate of recurrence (95% CI 13.36–13.50) by postoperative year 10. One of the limitations of this study is the short follow-up duration, and additional investigation will be necessary to assess whether diastasis plication during robotic ventral hernia repair is associated with differences in recurrence rates in the long term. Additionally, practice patterns, level of training in robotic surgery, and patient characteristics may vary by institution, limiting the generalizability of our results. This study is also specific to rTAPP procedure and our findings may not extend to other robotic surgery techniques. Our data also lack patient-reported outcome measures, and most patients were followed for less than one year after the procedure.

Ultimately, the adoption of concurrent robotic repair of ventral hernia and rectus diastasis will depend not only on clinical evidence but also policies set by payors and options available to providers. Currently, this procedure is most commonly done in conjunction with existing ventral hernias and fixed and billed for as a part of a bundled hernia repair procedure. Correction of rectus diastasis without the existence of a ventral hernia has been categorized as a primarily cosmetic, rather than functional, procedure. As Rosen et al. observed in their systematic review of insurance coverage in the US, private insurance policies frequently do not cover diastasis repair with 78.4% of companies (*n* = 51) denying coverage in all cases regardless of symptoms [12]. In the absence of payment for rectus diastasis treatment, it is unclear how widely surgeons will incorporate concurrent diastasis plication with ventral hernia repairs as incentivizing surgical repair and further study of this condition will remain difficult. This study supports routine diastasis plication during robotic repairs of small ventral hernias as it is safe, efficacious, and does not increase surgical risk. Avenues for future investigation may include comparisons between rTAPP and other robotic techniques, long-term comparisons of hernia repair outcomes with and without diastasis plication, assessment of optimal mesh size, examination of cosmetic outcomes, and predictors of need for reoperation following rTAPP with diastasis plication.

## Conclusion

Repair of small ventral hernias with concurrent rectus diastasis plication can be performed safely and effectively via rTAPP without need for reoperation and with no evidence of clinical or radiographic recurrence. Routine diastasis plication with rTAPP in a case series of 30 patients at a single center demonstrates no observed increase in short-term complications or adverse events.
